# A Multiorgan Segmentation Model for CT Volumes via Full Convolution-Deconvolution Network

**DOI:** 10.1155/2017/6941306

**Published:** 2017-09-17

**Authors:** Yangzi Yang, Huiyan Jiang, Qingjiao Sun

**Affiliations:** Software College, Northeastern University, Shenyang 110819, China

## Abstract

We propose a model with two-stage process for abdominal segmentation on CT volumes. First, in order to capture the details of organs, a full convolution-deconvolution network (FCN-DecNet) is constructed with multiple new unpooling, deconvolutional, and fusion layers. Then, we optimize the coarse segmentation results of FCN-DecNet by multiscale weights probabilistic atlas (MS-PA), which uses spatial and intensity characteristic of atlases. Our coarse-fine model takes advantage of intersubject variability, spatial location, and gray information of CT volumes to minimize the error of segmentation. Finally, using our model, we extract liver, spleen, and kidney with Dice index of 90.1 ± 1%, 89.0 ± 1.6%, and 89.0 ± 1.3%, respectively.

## 1. Introduction

Precision medicine was firstly raised in 2011 and has continued developing the new medical model: biologically informed therapies [1] and computer-aided diagnosis. As we know, precise organ segmentation relies on computer-aided diagnosis necessarily. Due to the fact that segmenting organ manually is time-consuming, material-consuming, and labor-intensive, many methods are proposed for the auto-segmentation of individual organs in previous work [2–7]. However, when it comes to multiorgan segmentation, the problems left to address are not only the variability of the shape and position of abdominal organs, but also the complicated interrelations among the organs, which make segmentation become a challenge.

In order to segment multiple organs accurately, several approaches have been proposed. Okada et al. analyzed organ correlations and prior information of shapes to improve accuracy of multiorgan segmentation [8]. Shimizu et al. used an abdominal cavity standardization process and atlas guided segmentation with parameters estimated by EM algorithm [9]. Oda et al. selected similar atlas as the input image for segmentation and refine the results based on graph cut [10]. Chu et al. proposed an automated multiorgan segmentation method based on a scale hierarchical probabilistic atlas [11]. However, because of the variety of organs among patients, the important sophisticated information of special atlas may be ignored in the above methods.

Recently, Convolutional Neural Network (CNN) proves itself at visual recognition [12–14]. Simonyan and Zisserman increased depth of networks, which showed a significant improvement for image recognition [15]. Additionally, CNN can generate a good deal of valuable consequence in semantic segmentations [16–19]. Hong et al. raised a novel decoupled architecture with heterogeneous annotations to classification and segmentation networks separately [20]. Recent approaches in CT images segmentation are mainly driven by CNN. Roth et al. used multilevel deep convolutional networks for pancreas segmentation [21]. Zhen et al. employed a structure including multiscale deep networks and random forests for direct estimation of cardiac ventricular volumes [22]. However, these works segmented organs simply based on local features, each layer may lose information (especially the location of the organs) after convoluting, and the size of input images is limited as well. Fully convolutional network (FCN) converts an existing CNN architecture by changing all fully connected layers to convolutions and adding deconvolutional layers in network [23–26]. Compared to the previous CNNs researches, they accept any size of images as input and the labels are predicted in pixel level. Although these networks can extract the overall shape of objects, intensity and spatial features are ignored when segmenting organs.

Therefore, in order to take into account variability information and deeper correlations of abdominal organs, we employ a new model for multiorgan segmentation. Our contributions are as follows:

1. We propose a full convolution-deconvolution network (FCN-DecNet) with new architecture which includes deconvolutional layer, new unpooling layer, and fusion layer. So, in multiorgan segmentation process, our network can extract different scales of shape details and record location information within the feature maps.
2. To overcome the lack of spatial information and gray values of the segmentation regions in FCN-DecNet, we construct a multiscale weights probabilistic atlas (MS-PA), which integrates atlas information as well as rough segmentation results from FCN-DecNet.
3. We propose a method to optimize FCN-DecNet by combining segmentation results of FCN-DecNet with MS-PA and take the ensemble as the final segmentation results.

The rest of this paper is organized as follows. In Section 2, we describe our new multiorgan segmentation model with the details of the FCN-DecNet and MS-PA, respectively. To verify our method, experimental results are compared and a general discussion regarding our approach is done in Section 3. Lastly, conclusions are summarized in Section 4.

## 2. Methods

### 2.1. Overview

**Figure 1:**
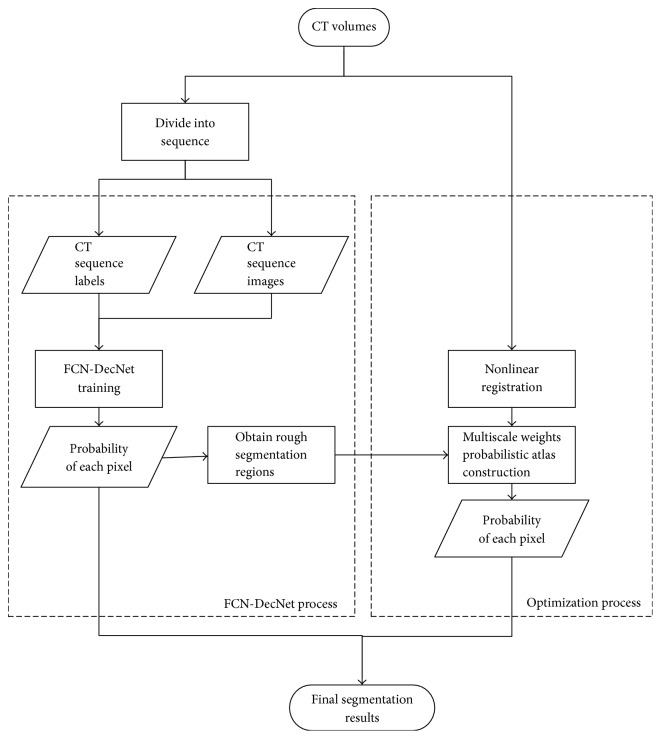
Flowchart of our proposed model.

In this paper, we construct a network for multiorgan segmentation, further optimized with MS-PA. A brief summary of the basic flow is described in Figure 1. Before the whole segmentation process, we divide CT volumes into sequence slices as the input of FCN-DecNet. In FCN-DecNet segmentation process, a network with multiple layers is trained. Probability of each pixel is obtained from output and taken as the rough segmentation results. In optimization process, we first take CT volumes and rough segmentation results from FCN-DecNet as the input of MS-PA to generate a multiscale weights probabilistic atlas. Then, probability of each voxel is calculated through Bayes-based estimation and used to refine rough segmentation results; in this way, final segmentation is gotten.

### 2.2. FCN-DecNet Network for Segmentation

#### 2.2.1. Architecture

**Figure 2:**
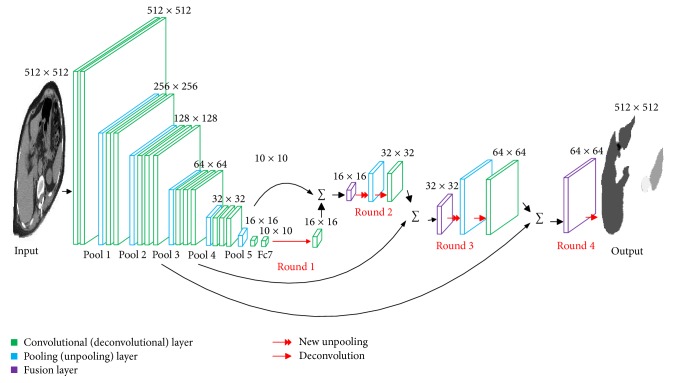
The detailed architecture of our networks. The processes in each round are shown as red color, including new unpooling and deconvolution. The concrete classes of organs are acquired by FCN-DecNet.

Our network is particularly composed of five parts: convolutional layer, pooling layer, deconvolutional layer, new unpooling layer, and fusion layer.  Figure 2 shows the architecture of FCN-DecNet in this paper. The network is initialized from the VGG-16 net [15], we discard the final classifier layer and convert all fully connected layers to convolutional layers, and then we append a 1 × 1 convolution with channel dimension 4 to predict scores of liver, spleen, kidney, and background after pool 3, pool 4, pool 5, and fc7. Since unpooling and deconvolution have different contributions to reconstructing the original size of segmentation regions, unpooling records the original locations; deconvolution tends to capture detail shapes. 4 rounds of unpooling and deconvolution in FCN-DecNet are followed for upsampling the coarse outputs. Based on the enlarged coarse outputs in first round, the second and third rounds reconstruct the location and shape of the organs. Meanwhile, from the first to the third round, the outputs are fused with the corresponding prediction scores at pool 3, pool 4, and pool 5. Considering the little improvement for the even lower layer fusion, in the fourth round, we directly deconvolve and get the output probability with 8 pixels' stride at final layer.

#### 2.2.2. New Unpooling

Unpooling is an approximate inverse of max pooling operation by recording the value and location of the maximum activation during pooling and filling maximum value back as well as setting other activation to zero within each pooling region. However, zero values make difference becoming large between max activation and other activation within each pooling region, which would increase intersubject variance in local segmentation regions. To deal with this issue, our new unpooling fills values at other activation after comparing maximum activation value with mean value of the whole map instead of just filling zero. Therefore, the generated segmentation organs from the above layer are placed into the appropriate locations and the shapes of organs in this layer are also preserved at the same time. The mean value of the whole map is calculated when yielding pooled maps and filled in vacancy during unpooling. The mathematical form is defined as

(1) $$\begin{array}{c} f\left(\;v\;\right)=\left\{\begin{array}{cc} u & \text{if }v\geq u\\ 0 & \text{otherwise,} \end{array}\right. \end{array}$$

where *f*(*v*) is filling function; *u*, *v* denote mean value of the whole map and maximum activation value during pooling, respectively.

**Figure 3:**
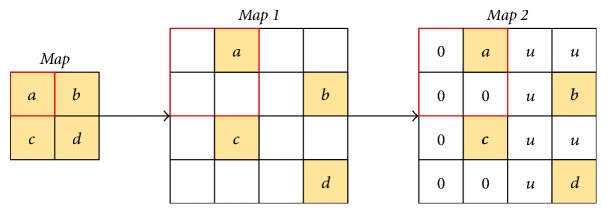
Illustration of new unpooling process.

The detail of unpooling is illustrated in Figure 3, where *a*, *c* < *u* ≤ *b*, *d*. The output of an unpooled map is first enlarged to *map*  1 after filling maximum activation values *a*, *b*, *c*, *d* in the same location. Then, for each of the maximum activation values, if they are larger than *u*, other activation within corresponding pooling region is filled with *u*; otherwise it is filled with 0. In this way, *map*  1 is changed to *map*  2 and taken as the final output of the new unpooling. Meanwhile, deconvolution operation is employed to densify *map*  2 because vacancy still exists sparsely when *v* < *u*.

#### 2.2.3. Analysis of FCN-DecNet

**Figure 4:**
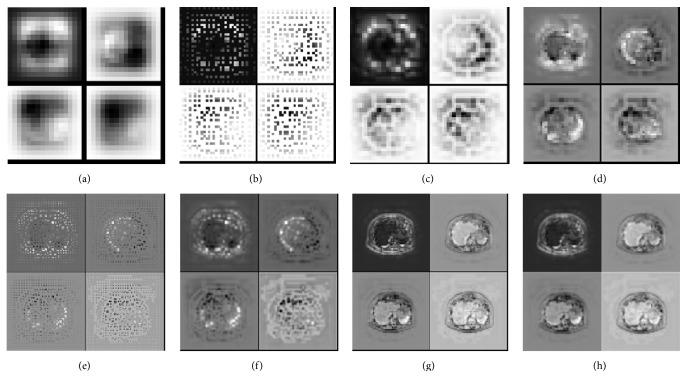
Visualization of activation in our network. We select most representative activation maps for net visualization; (a) fuse1 layer (16 × 16), (b) new unpooling layer (32 × 32), (c) deconvolutional layer (32 × 32), (d) fuse2 layer (32 × 32), (e) new unpooling layer (64 × 64), (f) deconvolutional layer (64 × 64), (g) fuse3 layer (64 × 64), and (h) deconvolutional layer (512 × 512).

*(A) Net Visualization*. Net visualization represents the input pattern that stimulates the given feature map in the model accurately [23]. We can judge the reasonableness of design structure and understand specific roles of deconvolution and unpooling processes by observing corresponding activation maps, as shown in Figure 4. From round 1 to round 4, coarse to fine segmentation regions are reconstructed through unpooling and deconvolution. Unpooling reconstructs the rough shapes of the organs at appropriate location. Deconvolution learns multiple filters to densify segmentation regions obtained from the previous unpooling layer. Furthermore, through a series of fuse operations, class-specific patterns are found from lower layers to higher layers.

**Figure 5:**
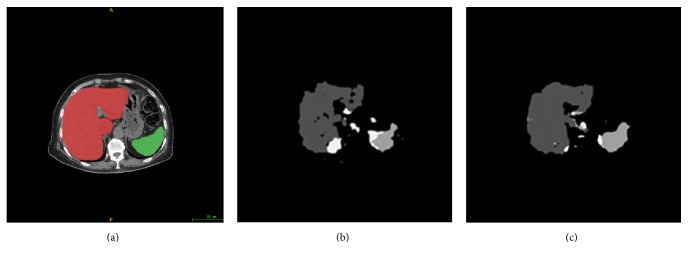
Comparison of segmentation results between FCN-8s and FCN-DecNet. (a) Input images, (b) FCN-8s, and (c) FCN-DecNet.

*(B) Net Comparison*. There are three kinds of output from FCN fusing information from layers and upsampling with different strides: FCN-8s, FCN-16s, and FCN-32s. And FCN-8s net shows the highest performance among them [24]. So we make comparison with FCN-8s and get segmentation results in Figure 5. Discovering our net not only fills vacancy area of FCN-8s but also improves the accuracy when reconstructing the boundary of organ. However, small scattered regions are still not belonging to organs. So the MS-PA is applied to FCN-DecNet for more precise results.

### 2.3. FCN-DecNet Optimization

#### 2.3.1. MS-PA Construction

Our method performs segmentation of three abdominal organs (liver, spleen, and kidney) in target CT volume. Before the optimization, MS-PA is first constructed to obtain organ regions, which provides a priori information about the intensity and spatial information of the target CT volume. All atlases are aligned by nonlinear registration (affine transformation and B spline transformation) to reduce location and shape variation of organs among different patients. The label spaces of organs are manually marked by doctors, denoted as *L* = {*l*_0_, *l*_1_, *l*_2_, *l*_3_} (represent background, liver, spleen, and kidney, resp.).

In order to segment different organs, more comprehensive information is needed. We give a hierarchical weight model, including three scales (image-wise, organ-wise, and voxel-wise). Here, we take the similarity between reference atlases in *I* = {*I*_1_,…, *I*_*m*_,…, *I*_*n*_} (*n* is the number of atlases; *I*_*m*_ is one of the atlases in *I*) and target CT volume *S* as image-wise weight *W*_*am*_ and the similarity between organs in *S* and *I*_*m*_ as organ-wise weight *W*_*om*_. Then, in each voxel at the same position between *S* and *I*_*m*_, we calculate the similarity of region composed of 242 adjacent voxels (9 × 9 in previous slice, 9 × 9 in next slice, and 9 × 9 − 1 in current slice) as voxel-wise weight *W*_*vm*_. *W*_*am*_ is used to select the most similar atlas in *I* after registration, so we use Pearson correlation coefficient to calculate. Meanwhile, we divide *W*_*om*_ into two parts: one part is to compute rough overlapping proportions between organs in *S* and *I*_*m*_; the other part is to further rectify inaccurate places. Therefore, Jaccard index and Pearson correlation coefficient are used to calculate, respectively. As for *W*_*vm*_, we use normalized Euclid distance directly to calculate intensity relevance among voxels. They are successively defined by

(2) Wam=PCCS,Im,Wom=JISo,Imo∗PCCSo′,Imo,Wvm=1−EUDSv,Ivmmax⁡EUDSv,Ivm,

where *I*_*mo*_ and *S*_*o*_ are the organ regions in *I*_*m*_ and corresponding positions in *S*; particularly, *S*_*o*_ is obtained from the rough segmentations of FCN-DecNet; *S*_*o*_′ is the region in *S* with same position in *I*_*mo*_. *I*_*vm*_ and *S*_*v*_ are adjacent regions of voxel with same position in *I*_*m*_ and *S*. PCC, JI, and EUD indicate Pearson correlation coefficient, Jaccard index, and Euclid distance, respectively, PCC and JI are defined by

(3) PCCX,Y=CovX,YVarXVarY,JIX,Y=X∩YX∪Y,

where Cov(*X*, *Y*) is the covariance of *X* and *Y*. Var(*X*) and Var(*Y*) are the variances of *X* and *Y*. And the probability of each voxel *p* belonging to organ *l* is calculated by

(4) Pl=∑mWamWomWvmfl,l′∑mWamWomWvm,fl,l′=1if  l=l′0otherwise.

The voxel level probability result of MS-PA is obtained through Bayes-based estimation that is defined by

(5) $$\begin{array}{c} p\left(\;l\;|\;V_{m}\;\right)=\frac{P\left(V_{m}\;|\;l\right)P\left(l\right)}{P\left(V_{m}\right)}, \end{array}$$

where intensity distribution of each organ *P*(*V*_*m*_∣*l*) is a normal with parameters estimated by EM algorithm, forming the Gaussian mixture distribution of the whole atlases. Based on the joint probability of all organs, *P*(*V*_*m*_) = ∑_*l*_*P*(*V*_*m*_∣*l*)*P*(*l*) is calculated.

**Figure 6:**
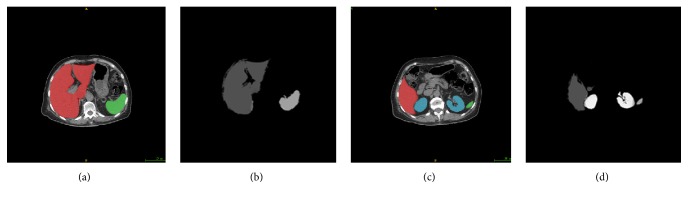
Representative slices in the results of MS-PA are selected as examples. (a) and (c) are original images and the true boundaries manually defined by doctor (red, green, and blue regions show ground truth of liver, spleen, and kidney in sequence). (b) and (d) are corresponding results.

Figure 6 shows the segmentation results from MS-PA, which highly depend on the spatial information and gray values of the organs. However, since the limitation number of atlases in *I*, irrelevant voxels especially around the boundary of organs may be classified in error.

#### 2.3.2. Optimization through MS-PA

Our FCN-DecNet obtains finer segmentation results than FCN-8s. Yet, inaccurate segmentation regions still appeared. So in optimization step of FCN-DecNet, a Bayes-based estimation through MS-PA is deployed to refine the rough segmentation. All pixel level probabilities of FCN-DecNet in one volume are first stacked into voxel level probabilities. Then we calculate the mean value of both voxel probabilities in FCN-DecNet and MS-PA. In this way, the best matching label *l* is estimated by maximum mean voxel probabilities. By the amelioration of MS-PA, our model considers high-scale shapes and locations information of organs in FCN-DecNet as well as the spatial information and gray values of the segmentation regions in optimization step.

## 3. Results and Discussion

**Figure 7:**
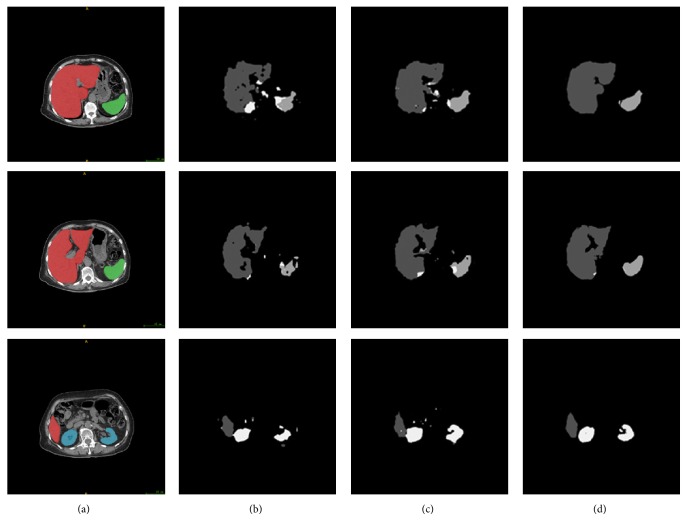
Representative slices of organs segmentation results on CT volumes. We further improve performance significantly through the FCN-DecNet + MS-PA. (a) Input images, (b) FCN-8s, (c) FCN-DecNet, and (d) FCN-DecNet + PA.

**Figure 8:**
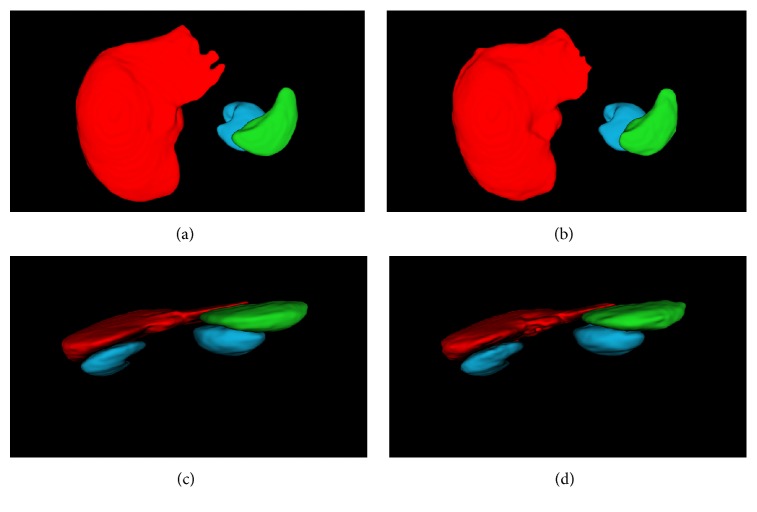
3D view of segment organs based on our method. (a) and (c) are ground truth; (b) and (d) are our segmentation results. Red, green, and blue indicate the liver, spleen, and kidney, respectively.

We evaluate our segmentation algorithm with 3D abdominal CT volumes of 12 patients, and each volume contains altogether about 70 abdominal slices from the same CT scanner. To offset insufficient data and select available model, 4-fold cross validation is applied with the data randomly split into training and testing sets. All the organ boundaries are manually defined by us and approved by a doctor. CT volumes are divided into slices with fixed-size 512 × 512 for training FCN-DecNet, and then volumes themselves are taken as input in MS-PA construction as well. In addition, we reconstruct organs using the sequence of segmentation results. Considering the best results from the FCN-8s architecture in [24], Figure 7 gives the segmentation results from FCN-8s, FCN-DecNet, and FCN-DecNet + MS-PA. Compared to FCN-8s, the vacancy in organs is filled and the boundary is smoothed through FCN-DecNet, which not only builds overall shape of an object, but also corrects wrong segmentation results from FCN-8s. Moreover, the training time is decreasing based on our efficient net. Although our results are better than FCN-8s on account of holding the shapes and locations of the organ, inaccurate segmentations still exist. So we incorporate with spatial information as well as gray values and can find that the false segmentation regions are eliminated and the overshapes of reconstructed organs are close to ground truth through FCN-DecNet + MS-PA in Figure 7.  Figure 8 shows 3D view of the segmentation results compared with ground truth.

Then, we make comparison between FCN-DecNet and FCN-8s in Table 1 via indicators defined in [24]. We see that the pixel and mean IU accuracy of FCN-DecNet seem close to FCN-8s, but mean accuracy is improved substantially in FCN-DecNet.

In order to further evaluate our method, Table 2 reports maximum segmentation accuracies in 4-fold cross validation using Dice similarity index [29, 30], Precision, and Recall rate, defined by

(6) Dice=2TP2TP+FP+FN,Precision=TPTP+FP,Recall=TPTP+FN,

where TP, FP, and FN denote the number of voxels relevantly classified, the number of voxels irrelevantly classified, and the number of voxels in ground truth that is ignored. Accuracies are rising via cross validation. This further explains that FCN-DecNet + MS-PA may be highly generalizable in cross-dataset assessment.

We use the Dice similarity index which measures the volume overlap between the ground truth and segmentation results. In comparison with state-of-the-art methods as reported in Table 3, our method performs better. Specifically, the segmentation accuracy is improved compared to Shimizu et al. [9], Liu et al. [27], and Okada et al. [8] with similar training samples. For others, our maximum segment Dice values of organs are close to Chu et al. [11] and Wolz et al. [28] but lower than them in general. This is because our cases are much less than Chu et al. [11] and Wolz et al. [28], which approximately equal 100 cases. What is more, data variation in nonlinear registration may bring influence to the effectiveness of the results. For instance, the size of abdominal cavity of some patients is too big or too small.

## 4. Conclusion

This paper proposes a new coarse-fine model for multiorgan abdominal segmentation (liver, spleen, and kidney). A new fully convolutional network (FCN-DecNet) is trained and refined in optimization step. For our FCN-DecNet, new unpooling layer records locations of organs, deconvolutional layer reconstructs detailed shapes of organ, and fusion layer is employed to make local predictions which make use of correlations among regional pixels. The learning process of FCN-DecNet is shown via visualizing the corresponding activation maps. For optimization step, three scales (image-wise, organ-wise, and voxel-wise) are considered in construction with multiscale weights probabilistic atlas (MS-PA), and Bayes-based estimation through MS-PA is employed to improve the coarse segmentation results of FCN-DecNet further. The proposed method shows better segmentation performance compared to other methods with similar number of cases. Further perfection will be done in future work, including evaluating more organs and using large number of cases to train our model.

## Figures and Tables

**Table 1:** Comparison of FCN-8s and FCN-DecNet is shown with pixel accuracy, mean accuracy, and mean IU accuracy.

Method	Pixel acc. (%)	Mean acc. (%)	Mean IU (%)
FCN-8s	98.7	80.5	69.2
*FCN-DecNet*	*98.6*	*91.4*	*70.0*

**Table 2:** 4-fold cross validation: maximum segmentation results by FCN-DecNet + MS-PA.

Organ	Dice (%)	Precision (%)	Recall (%)
Liver	91.1	95.3	87.3
Spleen	91.6	95.0	95.6
Kidney	90.3	93.9	86.6

**Table 3:** Comparing segmentation accuracy with other methods based on Dice similarity index.

Method	Cases	Dice similarity index (%)			
		Liver	Right kidney	Left kidney	Spleen
*Proposed*	*12*	*90.1 ± 1*	*89.0 ± 1.3*		*89.0 ± 1.6*
Shimizu et al. [9]	10	89.0	85.0	78.0	84.0
Liu et al. [27]	12	77.0	74.0	73.0	69.0
Okada et al. [8]	28	89.1	88.2	87.4	82.5
Chu et al. [11]	100	95.1 ± 1	90.1 ± 5		91.4 ± 5.7
Wolz et al. [28]	100	94.4	94.3		90.9
